# Study on the Effect and Application Value of Heat-Inactivated Serum on the Detection of Thyroid Function, Tumor Markers, and Cytokines During the SARS-CoV-2 Pandemic

**DOI:** 10.3389/fmed.2021.742067

**Published:** 2021-10-18

**Authors:** Enjun Xu, Tao Li, Qiuli Chen, Zhongxin Wang, Yuanhong Xu

**Affiliations:** Department of Clinical Laboratory, First Affiliated Hospital of Anhui Medical University, Hefei, China

**Keywords:** SARS-CoV-2, test indicator, cytokines, pandemic, serum inactivation

## Abstract

**Purpose:** The current explored the impact of heat inactivation of blood samples on the results of a particular clinical test and its potential application value during the SARS-CoV-2 pandemic. We have aimed at providing a reference for clinical testing methods during the pandemic.

**Methods:** Blood samples were selected from our department's routine clinical examination between January 2021 and June 2021. The levels of these samples for quantitative detection of these indicators in each group (*n* = 90 cases/group) covered normal reference ranges and medically determined levels. For qualitative testing of the indicators, the specimens were additionally classified as negative, weakly positive, and positive (*n* = 20 cases/group). The specimens were then inactivated, and the differences in relevant indicators before and after inactivation were evaluated.

**Results:** A statistically significant difference was evident between the levels of TSH, T3, FT4, FT3, AFP, NSE, CYFRA211, IRI, IL-1β, IL-6, IL-8, IL-10, IL-2R, and TNF-α in the non-inactivated group 1 and the inactivated group 1 (*P* < 0.05). Among them, there was a strong correlation between TSH, T3, FT4, FT3, CYFRA211, IRI, IL-1β, IL-6, IL-8, and IL-2R levels in the two groups (*P* < 0.05), however, there was no correlation between AFP (*P* = 0.256) and NSE (*P* = 0.352) levels between the two groups (*P* > 0.05). The detected values of low-level AFP (<4 ng/mL), IL-10, and TNF-α after inactivation were all lower than the detection limit. There was not any statistically significant difference in the levels of tumor markers, such as CEA, CA125, CA724, CA199, CA153, and the quantitative levels of T4, Vit. D, HCG, CPS, and five items of hepatitis B virus (*P* > 0.05). The positive rate of anti-nuclear antibodies after inactivation was not statistically different from the ones observed before inactivation (*P* > 0.05). Upon correction by the regression equation, the observed levels of TSH, T3, FT4, FT3, CYFRA211, IRI, IL-1β, IL-6, IL-8, and IL-2R were not significantly different from those before inactivation (*P* > 0.05).

**Conclusion:** The heat inactivation of blood samples had different various effects on different test indicators, and some indicators could be corrected by employing regression equations. This detection method could potentially be employed during the SARS-CoV-2 pandemic, thereby effectively preventing iatrogenic infections.

## Introduction

The Severe Acute Respiratory Syndrome-related Coronavirus 2 (SARS-CoV-2) is the B subgroup of *Betacoronavirus* and is essentially an enveloped single-stranded positive-stranded RNA virus (GenBank No.MN908947). It possesses a helical structure and is highly infectious (1–3). At the moment, it is known that SARS-CoV-2 spreads by respiratory droplets, close contact, and prolonged exposure to high aerosol concentrations (4–6). The majority of the Coronavirus Disease 2019 (COVID-19) patients suffer from co-morbidities and underlying disorders, such as tumors, cardiovascular diseases, thyroid, and hepatitis B virus. In addition to SARS-CoV-2 nucleic acid detection and antibody detection, routine hematology indicators are fundamental for clinical typing as well as for the comprehensive treatment and monitoring of patients (7, 8). SARS-CoV-2 is highly infectious, which exposes the investigators and clinicians to great risks and challenges during their efforts for the prevention and control period of the pandemic, especially during routine clinical inspections of confirmed and suspected patients (9, 10). According to GB19489-2008 “General Requirements for Laboratory Biosafety,” relevant laboratory tests for patients with COVID-19 should be carried out in a biosafety tertiary laboratory (BSL-3). However routine clinical laboratory testing is limited by the level of protection and limited space. As a potential solution to this problem, if the patient's blood sample is centrifuged and inactivated in the BSL-3 laboratory before testing, then the inactivated blood or serum sample can be tested in a routine laboratory. Such inactivation would not only eliminate the need for sample testing in BSL-3 laboratory conditions but also greatly substantially reduce the risk of infection of the laboratory personnel handling the specimen (11). There have been quite a few reports in the literature on the impact of exposing a blood sample to 56°C for 30 min, on the analysis of SARS-CoV-2 nucleic acid, routine biochemistry, blood routine, and coagulation detection. However, the influence of 30 min inactivation treatment at 56°C upon the detection of tumor markers, thyroid function, cytokines, and autoantibodies is rather ambiguous. Herein, we investigated whether the inactivation treatment at 56°C for 30 min had any influence on the evaluation of relevant blood samples.

## Materials and Methods

### The Source of the Specimen

The blood specimens were obtained from routine clinical examination specimens in our department. Quantitative detection indicators were collected based on high-, medium-, and low-value (*n* = 60 cases), and the remaining 30 cases were used for post-calibration detection. Quantitative detection indicators were collected based on negative, weakly positive, and positive (*n* = 60 cases). The samples chosen were all fresh specimens taken on the same day, ensuring a detection gap of no more than 2 h between specimens before and after inactivation. The testing system utilized was run strictly in accordance with the relevant ISO15189 quality management system requirements, and its precision is higher than the relevant standards indicated in the manufacturer's statement or the relevant CLIA '88 recommendations. Identical fresh reagents were used to evaluate the specimens daily before and following inactivation as well as for interior quality control (IQC). If the IQC fails or there is a significant deviation from the mean value of the IQC, the quality control products were tested continuously at two concentration levels for 20 times in accordance with the requirements of NCCLS EP15-A, and the intra-batch imprecision and total precision of the two concentrations were calculated, respectively. Only when the findings were satisfactory, we would begin testing specimens on the same day before and after inactivation (Table 1).

**Table 1:** The mean levels and CVs of Internal Quality Control (IQC) and value ranges of these indicators.

**Indicators**	**Control Lot. (level 1, level 2)**	**IQC mean levels**		**IQC CVs(%)**		**Value ranges**
		**level 1**	**level 2**	**level 1**	**level 2**	
TSH (mIU/L)	40,371, 40,373	0.364	32.917	3.77	3.25	0.2–51.01
	40,391, 40,393	0.368	32.877	4.08	4.22	
T4 (nmol/L)	40,371, 40,373	58.39	214.45	6.20	4.59	4.7–368.3
	40,391, 40,393	67.93	176.17	7.07	5.59	
T3 (nmol/L)	40,371, 40,373	1.30	5.40	7.69	5.74	0.63–7.23
	40,391, 40,393	1.27	5.32	7.87	5.26	
FT4 (pmol/L)	40,371, 40,373	9.59	42.73	6.99	6.08	2.13–51.93
	40,391, 40,393	9.84	35.55	7.11	7.82	
FT3 (pmol/L)	40,371, 40,373	4.02	17.12	5.22	4.38	0.3–26.7
	40,391, 40,393	4.08	17.22	4.41	5.28	
AFP (ng/ml)	54,671, 54,673	9.98	235.06	4.91	4.24	0.61–446.6
CEA (ng/ml)	54,671, 54,673	2.74	66.14	5.47	3.19	0.22–1904
CA125 (U/ml)	54,671, 54,673	25.20	194.72	5.24	5.31	3.72–1775
CA724 (U/ml)	54,671, 54,673	4.38	39.59	3.65	3.64	0.2–245.9
CA199 (U/ml)	54,671, 54,673	21.79	224.00	4.77	3.89	3.12–3097
CA153 (U/ml)	54,671, 54,673	22.39	119.78	5.60	5.25	3.17–45.38
NSE (ng/ml)	54,671, 54,673	11.41	75.33	4.33	4.77	7.86–96.39
CYFRA21-1 (ng/ml)	54,671, 54,673	6.86	119.78	3.21	2.87	2.19–17.83
VitD (nmol/L)	64,911, 64,913	15.6	91.3	7.53	6.89	7.7–35.1
HCG (mIU/ml)	40,371, 40,373	6.32	153.87	7.99	6.97	45.87–9104
	40,391, 40,393	11.16	235.82	7.32	5.53	
IRI (mU/L)	40,371, 40,373	15.55	191.32	5.98	6.07	6.47–300
	40,391, 40,393	16.57	170.04	6.70	6.73	
Cps (ng/ml)	40,371, 40,373	0.52	6.79	5.77	5.15	0.66–12.93
	40,391, 40,393	0.84	7.00	4.76	4.71	
HBs-Ag (IU/ml)	407,779,407,774	91.68	3.76	5.45	6.32	1.87–1732
HBs-Ab (IU/ml)	457,381, 457,380	89.78	<2.00	3.81	0	2.12–396.8
HBe-Ag (COI)	483,246, 477,333	12.68	0.87	6.36	10.89	0.08–14.78
HBe-Ab (COI)	491,396, 491,394	0.58	1.52	6.52	4.31	0.02–1.53
HBc-Ab (COI)	488,568, 488,565	0.53	2.28	6.71	4.55	0.01–2.32
IL-1β (pg/ml)	ILCO10036, ILCO20036	93.67	427	6.65	5.78	6.52–59.3
IL-6 (pg/ml)	ILCO10036, ILCO20036	94.56	578	7.32	6.45	4.35–135.8
IL-8 (pg/ml)	ILCO10036, ILCO20036	113.45	867.36	7.12	6.35	6.98–254
IL-10 (pg/ml)	LXPC10029, LXPC20029	25.78	246.76	9.98	6.54	7.21–58.6
IL-2R (U/ml)	ILCO10036, ILCO20036	423.67	1897.35	6.73	6.25	130–1990
TNF-α (pg/ml)	ILCO10036, ILCO20036	70.75	485.67	8.02	7.34	4.67–104

### Instruments and Reagents

The quantitative detections of tumor markers and five markers of hepatitis B virus were completed using the Roche e 602 automatic chemiluminescence immunoassay analyzer. Thyroid function, C-peptide, and IRI were estimated using Siemens ADVIA Centaur XP automatic chemiluminescence immunoassay analyzer. HCG was tested using Siemens Immulite 2,000 XPi. Detection of the Cytokines was made using Siemens Immulite 1,000 whereas Vit D was detected by Liaison XL automatic chemiluminescence immunoassay analyzer. The anti-nuclear antibody detection kit was purchased from Oumeng, Germany. The water bath equipment was provided by Beijing Fuyilian Medical Equipment Co., Ltd. Interior quality control (IQC) products: Bio-Rad tumor marker plus control for tumor markers, and Bio-Rad immunoassay plus control for thyroid function, C-peptide, IRI, HCG, and Bio-Rad specialty immunoassay control for Vit D, and Original quality control products of Roche for five markers of hepatitis B virus, and Siemens Immulite cytokine control for Cytokines.

### Specimen Processing Method

First, each serum sample was divided based on the value into two portions. One part was regarded as non-inactivated group 1, and the other part was inactivated group 1. Following detection, the serum samples of non-inactivated group 1 were collected in 2 ml EP tubes in the biosafety cabinet, numbered and labeled as inactivated group 1, and finally sealed with sealing film. The specimen from inactivated group 1 was heat-treated in a water bath at 56°C for 30 min, then removed and placed in a biosafety cabinet. After removing the samples to be restored to room temperature they were centrifuged and tested on the same machine and reagent as non-inactivated group 1. The remaining 30 specimens were used for calibration testing and were divided into two groups: one as the non-inactivated group 2, and the other as the inactivated group 2 which was exposed to heat inactivation at 56°C for 30 min. The operation steps were the same as above.

### Statistical Analysis

In this study, SPSS 26.0 software was used for statistical analysis. The measurement data conformed to the normal distribution, which was expressed by $\bar{\text{x}}$ ± s, and the comparison of the level difference of each index before and after inactivation was performed by a paired *t*-test. The χ^2^-test was used to compare the positive rate of anti-nuclear antibodies. The detection value after inactivation was used to reverse the detection prior to inactivation (true value) to calibrate the detection value of certain markers following inactivation. The detection level before and after heat inactivation was compared using Pearson correlation and regression analysis. The study's significance level was set to α = 0.05.

## Results

### Comparison of Detection Levels Before and After Inactivation

The levels of TSH, T3, AFP, NSE, CYFRA211, IRI, IL-6, IL-10, IL-2R, and TNF-α in inactivated group 1 were substantially low in comparison to those in non-inactivated group 1, while the levels of FT4, FT3, IL-1β, and IL-8 were significantly higher in the inactivated group, and the difference was statistically significant (*P* < 0.05). Among them, the deviation of TSH and T3 was small, which might be considered a clinically acceptable error. The detection of low-level AFP (<4 ng/mL), IL-10, and TNF-α following inactivation were all well below the lower limit of detection. For all other indicators, there was no statistically significant difference in the detection levels of other indicators (*P* > 0.05; Table 2).

**Table 2:** Comparison of the test results of the two groups before and after heat inactivation.

**Indicators**	**Non-inactivated group 1**	**Inactivation group 1**	**Bias (%)**	***t*-value**	***P-value***
	**(*n* = 60)**	**(*n* = 60)**			
TSH (mIU/L)	7.94 ± 1.03	7.39 ± 0.95	−6.47 ± 5.46	4.28	0.001
T4 (nmol/L)	72.55 ± 9.69	69.22 ± 9.25	−2.37 ± 12.63	1.752	0.085
T3 (nmol/L)	1.11 ± 0.16	1.06 ± 0.15	14.47 ± 16.20	3.307	0.001
FT4 (pmol/L)	18.08 ± 8.64	56.53 ± 22.63	220.17 ± 59.84	16.541	0.001
FT3 (pmol/L)	5.27 ± 3.49	8.45 ± 5.74	59.89 ± 32.10	9.555	0.001
AFP (ng/ml)	13.26 ± 37.17	6.30 ± 13.40	−44.96 ± 26.52	7.598	0.001
CEA (ng/ml)	85.35 ± 265.39	87.13 ± 294.91	−4.48 ± 5.34	0.37	0.712
CA125 (U/ml)	124.63 ± 261.58	125.01 ± 266.80	−1.41 ± 4.75	0.319	0.751
CA724 (U/ml)	15.78 ± 37.94	16.23 ± 40.06	2.12 ± 4.60	1.319	0.194
CA199 (U/ml)	69.57 ± 117.72	67.81 ± 117.93	−4.86 ± 5.57	1.832	0.073
CA153 (U/ml)	10.63 ± 2.47	11.42 ± 2.63	2.05 ± 1.38	1.469	0.062
NSE (ng/ml)	21.9 ± 16.64	0.87 ± 0.61	−94.93 ± 4.36	7.724	0.001
CYFRA21-1 (ng/ml)	5.75 ± 3.23	0.71 ± 0.16	−85.02 ± 5.65	10.032	0.001
VitD (nmol/L)	12.41 ± 4.28	14.05 ± 4.47	2.38 ± 2.51	0.537	0.514
HCG (mIU/ml)	285.72 ± 15.64	287.35 ± 16.27	2.51 ± 1.48	0.629	0.431
IRI (mU/L)	79.52 ± 76.99	1.39 ± 1.39	−197.89 ± 1.19	5.923	0.001
Cps (ng/ml)	7.38 ± 1.52	7.61 ± 1.58	1.52 ± 1.07	0.543	0.521
HBs-Ag (IU/ml)	649.28 ± 47.32	653.71 ± 49.06	7.38 ± 4.25	0.574	0.516
HBs-Ab (IU/ml)	41.63 ± 5.28	43.25 ± 5.41	2.54 ± 2.07	0.641	0.432
HBe-Ag (COI)	0.17 ± 0.05	0.14 ± 0.03	−0.06 ± 0.02	0.538	0.572
HBe-Ab (COI)	0.75 ± 0.14	0.77 ± 0.16	0.16 ± 0.04	0.642	0.437
HBc-Ab (COI)	0.24 ± 0.12	0.21 ± 0.09	−0.07 ± 0.02	0.549	0.527
IL-1β (pg/ml)	18.63 ± 12.35	33.01 ± 20.15	86.76 ± 58.20	11.709	0.001
IL-6 (pg/ml)	32.10 ± 36.61	14.87 ± 18.79	−56.28 ± 12.51	6.131	0.001
IL-8 (pg/ml)	53.93 ± 59.41	95.37 ± 113.52	59.88 ± 34.89	5.872	0.001
IL-10 (pg/ml)	23.85 ± 16.16	<5.00	-	-	-
IL-2R (U/ml)	796.48 ± 453.03	365.33 ± 13.79	−58.25 ± 13.79	15.686	0.001
TNF-α (pg/ml)	21.21 ± 21.59	<4.00	-	-	-

### Correction of Results of Some Test Items

**Figure 1:**
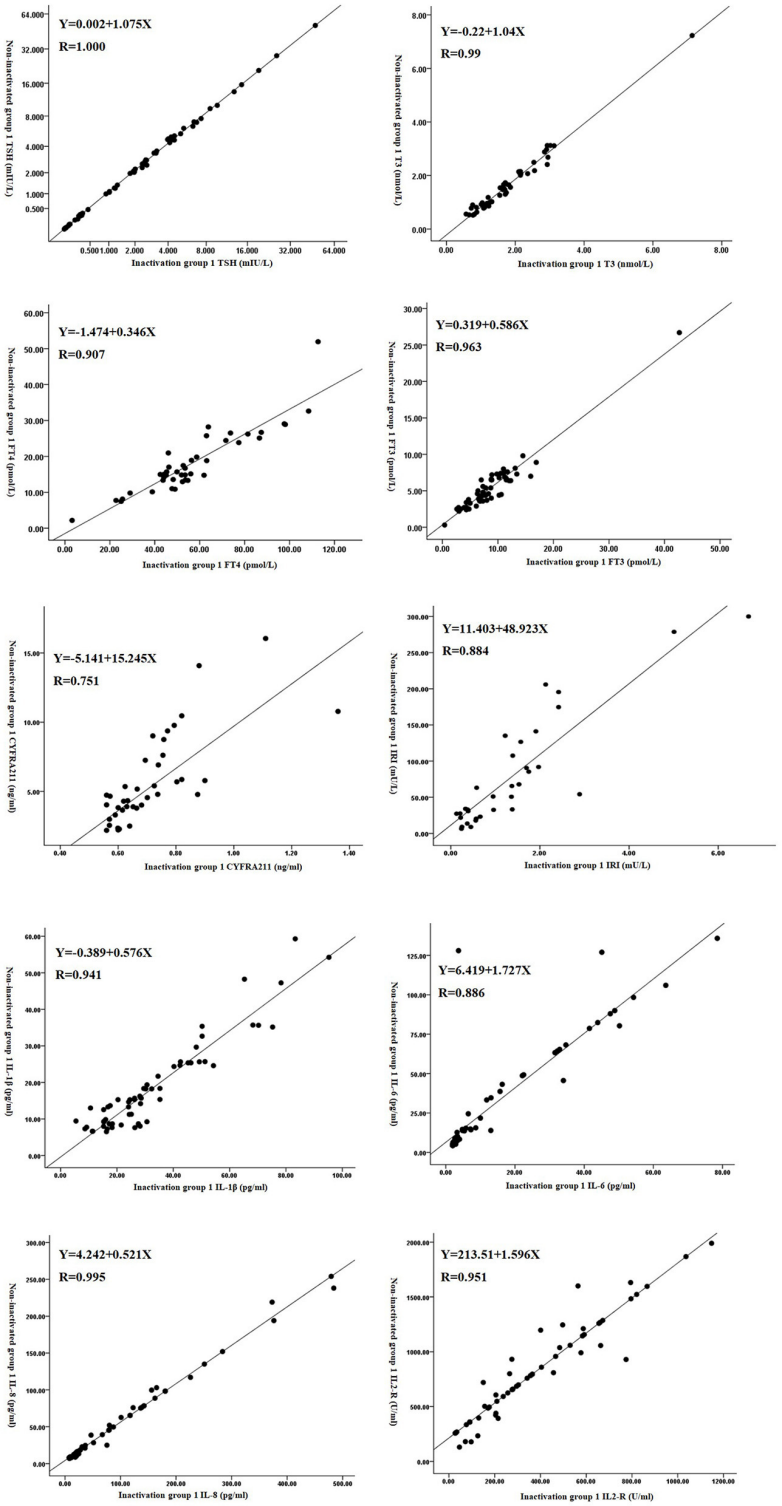
Scatter diagram of the detection levels before and after the inactivation of some test items.

The detection value after inactivation was used to calibrate the detection value of certain markers after inactivation by reversing the detection value of some items after inactivation. We used linear correlation and regression analysis to get the regression equation and correlation coefficient for the detection level before and after heat inactivation. We found that there was a correlation between TSH, T3, FT4, FT3, CYFRA211, IRI, IL-1β, IL-6, IL-8, and IL-2R between the two groups (*P* < 0.05). However, there was no correlation between the AFP (*P* = 0.256) and NSE (*P* = 0.352) levels of the two groups (*P* > 0.05, Figure 1 and Table 3).

**Table 3:** Correction equation for the results of some test items after inactivation.

**Indicators**	**Correlation coefficient r**	***P-value***	**Regression equation**	**Decisive factor**
				**r^**2**^**
TSH	1.00	0.001	*Y* = 0.002 + 1.075X	1.000
T3	0.99	0.001	*Y* = −0.22 + 1.04X	0.790
FT4	0.907	0.001	*Y* = −1.474 + 0.346X	0.822
FT3	0.963	0.001	*Y* = 0.319 + 0.586X	0.928
AFP	0.329	0.256	-	-
NSE	0.151	0.35	-	-
CYFRA211	0.751	0.003	*Y* = −5.141 + 15.245X	0.564
IRI	0.884	0.001	*Y* = 11.403 + 48.923X	0.781
IL-1β	0.941	0.001	*Y* = −0.389 + 0.576X	0.885
IL-6	0.886	0.001	*Y* = 6.419 + 1.727X	0.786
IL-8	0.995	0.001	*Y* = 4.242 + 0.521X	0.991
IL-2R	0.951	0.001	*Y* = 213.51 + 1.596X	0.905

### Comparison of Corrected Value and the True Value of Some test Items

We used the regression equation obtained from result 2.2 to correct the inactivated group 2. Compared with the level of non-inactivated group 2, the difference between the two groups was not statistically significant (*P* > 0.05; Table 4).

**Table 4:** Comparison of corrected values and true values of some test items ($\bar{\text{x}}$ ± s).

**Indicators**	**Non-inactivated group 2**	**After correction for inactivation group 2**	**Bias (%)**	***t*-value**	***P-value***
	**(*n* = 30)**	**(*n* = 30)**			
TSH (mIU/L)	7.94 ± 1.03	7.95 ± 1.05	0.26 ± 0.14	0.473	0.625
T3 (nmol/L)	1.11 ± 0.16	0.95 ± 0.18	−0.72 ± 0.43	0.639	0.421
FT4 (pmol/L)	18.08 ± 8.64	18.09 ± 8.67	0.17 ± 0.09	0.512	0.583
FT3 (pmol/L)	5.27 ± 3.49	5.32 ± 3.54	0.54 ± 0.12	0.612	0.437
CYFRA211 (ng/ml)	5.28 ± 3.27	5.38 ± 1.84	0.19 ± 0.40	0.197	0.846
IRI (mU/L)	93.47 ± 88.13	86.28 ± 80.75	0.29 ± 0.80	1.006	0.327
IL-1β (pg/ml)	17.26 ± 12.59	16.91 ± 10.06	0.08 ± 0.45	0.376	0.709
IL-6 (pg/ml)	38.45 ± 40.72	35.98 ± 35.15	0.27 ± 0.53	0.572	0.522
IL-8 (pg/ml)	57.52 ± 74.53	57.51 ± 73.91	0.09 ± 0.22	0.003	0.997
IL-2R (U/ml)	820.50 ± 505.187	820.67 ± 464.52	0.13 ± 0.40	0.005	0.996

### Comparison of the Positive Rates Before and After Anti-nuclear Antibody Inactivation

The variation in the positive rate of anti-nuclear antibodies before and after inactivation was not statistically significant in this study (*P* > 0.05; Table 5).

**Table 5:** Comparison of the positive rates before and after anti-nuclear antibody inactivation [*n* (%)].

**Inactivation**	**Result**	***n***	**Positive rate**	**x^**2**^**	***P-value***
**method**			**(%)**		
Non-inactivated group 1 (*n* = 60)	+	39	65.00	0.036	0.849
	-	21			
Inactivation group 1 (*n* = 0)	+	38	63.33		
	-	22			

## Conclusion

SARS-CoV-2 is sensitive to ultraviolet light and heat. Studies have shown that heating at 56°C for 30 min or exposure to ether, 75% ethanol, chlorine-containing disinfectant, peracetic acid, and chloroform can effectively inactivate the virus (12, 13). Laboratory staff can become infected after accidental extended exposure to aerosols at high concentrations when they come into contact with patients' blood, urine, feces, or other body fluids (14). Along with adequate biosafety protection, if the sample can be inactivated prior to testing without compromising the test result, the inspector's risk of infection can be significantly decreased. Additionally, this enables routine clinical testing of blood samples from COVID-19 patients in biosafety secondary laboratories, which will aid in the comprehensive treatment and prognosis monitoring of clinical COVID-19 patients. In this investigation, blood samples examined at our hospital were inactivated, and the changes in key indicators and the positive rate of detection of anti-nuclear antibodies were detected before and after inactivation. The purpose of this study was to increase the safety of serum sample testing during the pandemic and to minimize the risk of iatrogenic SARS-CoV-2 infection.

Recent studies have reported the influence of heat inactivation treatment upon the test results of routine clinical test indicators. Studies have shown (15–18) that following heat inactivation treatment, there is no statistical difference in the values of TP, TBIL, Cr, TC, TG, K+, Cl-, CRP, BUN, Glu, D-dimer, hemoglobin, white blood cells, platelet count, serological markers of infectious diseases, such as five indicators of hepatitis B virus, TP-Ab, and HIV. However, the values pertaining to some heat-labile enzymes, such as ALB, GLO, PA, AST, ALT, LDH, ALP, γ-GT, CK were found to be significantly reduced. Liu Changde et al. (19) showed that TCO2, GLO, TBIL, IBIL, HDL, PA, β2-MG, TP, ALB, and enzyme indicators were more affected by inactivation, while other conventional biochemical experience less difference. The disparity in study results was most likely driven by the disparity in detecting platforms and thermal treatment techniques. Several studies have also shown that (16, 20) inactivating blood samples at 56°C for 30 min had almost no effect on the results of SARS-CoV-2 antibody detection.

In this study, after the serum samples were inactivated at 56°C for 30 min, the levels of CEA, CA125, CA724, CA199, CA153, and other tumor markers, T4, VitD, HCG, CPS, five typical markers of hepatitis B virus, and anti-nuclear antibodies did not vary change significantly. However, the levels of TSH, T3, AFP, NSE, CYFRA211, IRI, IL-6, IL-10, IL-2R, and TNF-α were observed to be significantly reduced. This was most likely the effect of heat inactivation destroying the protein structure, resulting in reduced antigenicity. The levels of FT4 and FT3 increased significantly since presumably heat inactivation cause a part of T4 and T3 to be dissociated from thyroid-binding globulin. The levels of IL-1β and IL-8 were significantly increased, which **may be related to the exposure of some antigen epitopes after heat treatment**, and further studies are required to find out the specific reasons. Although the levels of TSH and T3 before and after inactivation were statistically different, the bias was relatively small and was within the clinical acceptable range. In this study, the levels of TSH, T3, FT4, FT3, CYFRA211, IRI, IL-1β, IL-6, IL-8, and IL-2R changed significantly. However, after correction by the regression equation, its level was not significantly different from that before inactivation. Therefore, clinical regression equation analysis could be performed on the indicators that had changed significantly after partial inactivation to reduce the difference caused by inactivation and improve the clinical value of the detected indicators.

The inactivation of blood samples had only minimal influence on the results of the clinical tests. Additionally, regression equations can be used to adjust for specific indications. This detection approach might be utilized successfully to restrict the spread of sample aerosols during the SARS-CoV-2 pandemic. This study successfully reinforced previous reports on the impact of serum inactivation upon tumor markers, thyroid function, IRI, Cps, cytokines, and anti-nuclear antibodies, completing the examination of the influence of serum inactivation on clinical tests findings. However, the study was not without limitations. For instance, the research was constrained by certain circumstances; the number of selected samples was limited, and large-scale data exploration was difficult. Future research should aim to increase the sample size as much as possible to derive more accurate findings. Simultaneously, the extinguishing detection technique needs enhancement. This technique has the potential to reduce the need for protective equipment and personnel and increase the efficiency of inspections, allowing it to be deployed on a broad scale during the SARS-CoV-2 pandemic when medical resources are scarce.

## Data Availability Statement

The original contributions presented in the study are included in the article/supplementary material, further inquiries can be directed to the corresponding author.

## Author Contributions

EX and TL contributed to conception and design of the study. EX and QC performed the statistical analysis and wrote sections of the manuscript. EX wrote the first draft of the manuscript. All authors contributed to manuscript revision, read, and approved the submitted version.

## Funding

Natural Science Research Project of Universities in Anhui Province (KJ2020ZD24).

## Conflict of Interest

The authors declare that the research was conducted in the absence of any commercial or financial relationships that could be construed as a potential conflict of interest.

## Publisher's Note

All claims expressed in this article are solely those of the authors and do not necessarily represent those of their affiliated organizations, or those of the publisher, the editors and the reviewers. Any product that may be evaluated in this article, or claim that may be made by its manufacturer, is not guaranteed or endorsed by the publisher.
